# A Study Protocol for the X-SIAGA Intervention: A Cluster Randomized Trial to Improve Household Preparedness for Disease X and Outbreaks in Selangor’s Indigenous Community

**DOI:** 10.7759/cureus.99216

**Published:** 2025-12-14

**Authors:** Ameerah S Abdul Shakor, Mariam Mohamad, Khalid Ibrahim, Izandis Mohamad Sayed

**Affiliations:** 1 Department of Public Health Medicine, Faculty of Medicine, Universiti Teknologi Majlis Amanah Rakyat (MARA), Selangor, MYS; 2 Ministry of Health Malaysia, Hospital Orang Asli Gombak, Kuala Lumpur, MYS

**Keywords:** community trial, epidemic, health education, health promotion, indigenous people, malaysia, orang asli, outbreak, preparedness, rural health

## Abstract

Introduction: Proactive preparedness strategies are more effective than reactive responses in minimizing the negative impacts of health emergencies, including outbreaks. Despite their potential to enhance community resilience, household preparedness initiatives are limited in Malaysia. The focus is primarily on flood disasters, often overlooking marginalized communities, such as indigenous groups. In Selangor, these communities live near forest edges and depend on the forest for livelihood, increasing their risk of exposure to zoonotic and novel threats such as Disease X. They also face challenges such as socioeconomic status, poor living conditions, healthcare barriers, and societal marginalization, making them particularly vulnerable to severe impacts from outbreaks. This study aims to evaluate the effectiveness of X-SIAGA, a newly developed intervention package, in improving household preparedness for Disease X and potential outbreaks among indigenous communities in Selangor, Malaysia.

Methods: This study will be a two-arm, parallel, single-blinded, cluster-randomized trial using multistage and simple random sampling to evaluate the effectiveness of X-SIAGA. The primary outcome is *Household Outbreak Preparedness* scores, with secondary outcomes including *Cognitive Preparedness *scores and *Preparedness Behaviour* scores. District and village clusters will be selected using multistage sampling and randomly assigned interventions via computer-generated numbers. Ninety-six households from eight villages are planned for recruitment, split evenly between the intervention and control groups. The intervention group will receive X-SIAGA along with educational brochures, while the control group will receive only the brochures. Outcome measures will assess changes from baseline to immediately after and one- and three-month post-intervention or control.

Results: As this is a study protocol, results are not yet available. The study is designed to assess changes in *Household Outbreak Preparedness* scores (primary outcome), as well as *Cognitive Preparedness scores* and *Preparedness Behaviour* scores (secondary outcomes).

Conclusion: This protocol describes an intervention study that will generate evidence on the effectiveness of X-SIAGA, a novel, culturally tailored, and theory-based intervention designed to improve household preparedness for Disease X and potential outbreaks among indigenous communities in Selangor.

## Introduction

The World Health Organization (WHO) introduced the term “Disease X” to denote a hypothetical yet potentially severe infectious disease that has not yet been identified as a cause of human disease but possesses the potential to trigger a global outbreak [1]. It is predicted to have a zoonotic origin and may be more deadly than COVID-19, imposing a significant burden on health systems, higher mortality, and major economic disruptions [2]. This impending public health threat necessitates proactive measures to mitigate its impact [3]. While eradication of future outbreaks is not realistically practicable, preparedness efforts can prevent them from escalating into health crises [4].

Preparedness strategies have been shown to be more efficient than crisis response measures in terms of limiting negative impacts, protecting human life, and mitigating financial consequences [5]. In addition, individual and household preparedness may enhance community resilience against disasters [6]. Thus, improving household preparedness is essential in managing disease outbreaks and reducing their negative impacts. However, preparedness efforts generally focus on macro-scale entities, such as healthcare systems and government bodies, often neglecting micro-scale levels like individuals, households, and local communities, who are first affected during such crises [7,8].

Preparedness programs at both community and individual levels remain limited globally. Moreover, existing initiatives tend to focus on raising awareness of diseases and natural disasters rather than implementing operational strategies to address outbreaks and emerging threats [4,7]. In Malaysia, community preparedness efforts are largely centered on flood disasters, with limited engagement of indigenous populations [9]. Existing programs primarily aim to raise awareness and reduce disease transmission risks; however, evidence suggests that such approaches do not necessarily translate into tangible preparedness outcomes [10,11]. To achieve preparedness, individuals should possess cognitive aspects of preparedness, including knowledge and positive attitudes, and should also demonstrate these through tangible behavior [12,13]. Preparedness programs should therefore not only focus on raising awareness and encouraging preventive actions but also equip individuals with guidance and skills for planning and reserving resources. Households that implement these measures may lessen the adverse impacts of health crises by enabling them to respond promptly until professional health support becomes available.

Global estimates indicate that between 50% and 92% of households are inadequately prepared for general emergencies [14]. In Hong Kong, a mere 3% of households are fully prepared for an influenza outbreak, while 41% exhibit no signs of preparedness actions [15]. There is a noticeable scarcity of data related to household outbreak preparedness in Malaysia. Local studies have noted that flood preparedness among Malaysians is relatively low, with less than half of the general public (44%) being adequately prepared and only 20% being fully prepared. Individuals primarily focus on protecting essential documents, planning for property safety, and locating the nearest evacuation facilities [16]. In contrast, flood preparedness levels in an indigenous community in Pahang are considerably lower, with 30% having property safety measures and only 10% implementing food and life-safety measures [9]. These findings indicate that both the general Malaysian population and marginalized communities exhibit inadequate preparedness levels; however, marginalized communities, especially indigenous groups, are more vulnerable and may experience more serious consequences during outbreaks, particularly with the threat of Disease X [17].

Indigenous communities in Malaysia are referred to as “Orang Asli.” Selangor has the third-largest Orang Asli population in Malaysia, consisting of 74% Proto-Malays ethnicity, with Temuan being the largest sub-ethnic group [18]. Numerous outbreaks of infectious diseases have affected the Orang Asli community, with many instances documenting cases of fatalities [19,20]. The higher risk of outbreaks among the Orang Asli can be attributed to various factors such as lower education levels, inferior socioeconomic status, poor living conditions, malnutrition, lower vaccine coverage, healthcare barriers, cultural challenges, and societal marginalization [9,19]. Preventive measures in Orang Asli communities have also been reported as inadequate [20].

Many Orang Asli villages in Selangor are located in deforested areas, and many continue to rely on the forest for their livelihood [9,18,19]. Therefore, the risk of human-wildlife contact cannot be discounted, emphasizing the need for targeted interventions for this group. Moreover, Selangor is one of the country’s most densely populated states, creating an additional threat. This setting increases the potential for rapid disease transmission beyond the Orang Asli villages, potentially leading to larger outbreaks and higher mortality rates if not effectively addressed. The COVID-19 pandemic demonstrated this impact when Selangor experienced the highest number of cases and fatalities attributable to its densely populated environment [21].

Considering these factors, the Orang Asli community in Selangor is highly vulnerable to severe impacts from outbreaks and novel threats such as Disease X. Targeted interventions are needed to improve their household preparedness, enabling them to reduce impacts while awaiting control measures from health authorities. Research-based evaluation of these interventions is essential to justify resource investment and support for marginalized groups and to propose adaptations for other communities and nationwide implementation to stakeholders.

To the best of our knowledge, there are no published studies that have examined tailored household preparedness interventions for outbreaks or Disease X among marginalized communities. This paper presents the research protocol for X-SIAGA (KesiapSIAGAan Penyakit X dan Wabak), an intervention package designed to enhance household preparedness for Disease X and potential outbreaks among the Orang Asli communities in Selangor, Malaysia. The trial will evaluate the effectiveness of the X-SIAGA intervention, in addition to standard educational brochures, compared with educational brochures alone, in improving household preparedness within this population.

The proposed hypotheses for this study are: (i) Hypothesis 1 (H1): The intervention changes household preparedness levels, potentially improving their capability to respond to Disease X and potential outbreaks; (ii) Hypothesis 2 (H2): The intervention changes cognitive aspects related to preparedness, improving knowledge and attitudes, including health beliefs, toward Disease X and outbreaks; and (iii) Hypothesis 3 (H3): The intervention changes behavioral aspects related to preparedness, enhancing skills and tangible preparedness practices among households for Disease X and outbreak preparedness.

To test the hypotheses, the objectives established for this study are as follows: (i) to assess the effectiveness of the intervention package on the level of *Household Outbreak Preparedness* among the Orang Asli in Selangor (primary outcome); (ii) to assess the effectiveness of the intervention package on the level of *Cognitive Preparedness *among the Orang Asli in Selangor (secondary outcome); and (iii) to assess the effectiveness of the intervention package on the level of *Preparedness Behaviour *among the Orang Asli in Selangor (secondary outcome).

## Materials and methods

Trial design

The study will employ a two-arm, parallel, single-blinded cluster randomized trial by using multistage sampling to evaluate the effectiveness of X-SIAGA in enhancing household outbreak preparedness against Disease X and outbreaks among the Orang Asli in Selangor. In this two-sided superiority trial design, the intervention group will receive the X-SIAGA intervention and educational brochures, while the control group receives only the brochures. The protocol adheres to the Standard Protocol Items: Recommendations for Interventional Trials (SPIRIT) guideline [22]. The SPIRIT schedule of enrollment, allocation, and assessments is shown in Table 1. 

**Table 1:** SPIRIT's schedule of enrolment, interventions, and assessments for X-SIAGA intervention. SPIRIT: Standard Protocol Items: Recommendations for Interventional Trials [22].

	Studt Period					
	Enrolment	Allocation	Post-allocation			
Timepoint	0	0	Baseline Assessment (T_0_)	Immediately Post-intervention (T_1_)	One-Month Post-intervention (T_2_)	Three-Month Post-intervention (T_3_)
Enrollment:						
Eligibility screen	X					
Informed consent		X				
Allocation		X				
Intervention:						
X-SIAGA intervention				X		
Standard educational brochure (control)				X		
Assessments:						
1^st^ questionnaire administration			X			
2^nd^ questionnaire administration				X		
3^rd^ questionnaire administration					X	
4^th^ (final) questionnaire administration						X

Theoretical and conceptual frameworks

This study applied the Social Cognitive Theory (SCT) and the Health Belief Model (HBM) to guide the development and implementation of the X-SIAGA intervention, aiming to influence the Orang Asli’s cognition, health beliefs, and behaviors related to household preparedness for Disease X and outbreaks. SCT is a widely used theory in disaster and emergency health preparedness programs, alongside the HBM. While the HBM focuses mainly on individual beliefs, SCT considers a wider array of factors, highlighting the interaction between behavioral, cognitive, and environmental influences in predicting behavior. Perceived self-efficacy, a cognitive aspect in SCT and part of individual health belief in the HBM, shapes how individuals view their environment and affects their intentions and actions [23].

**Figure 1:**
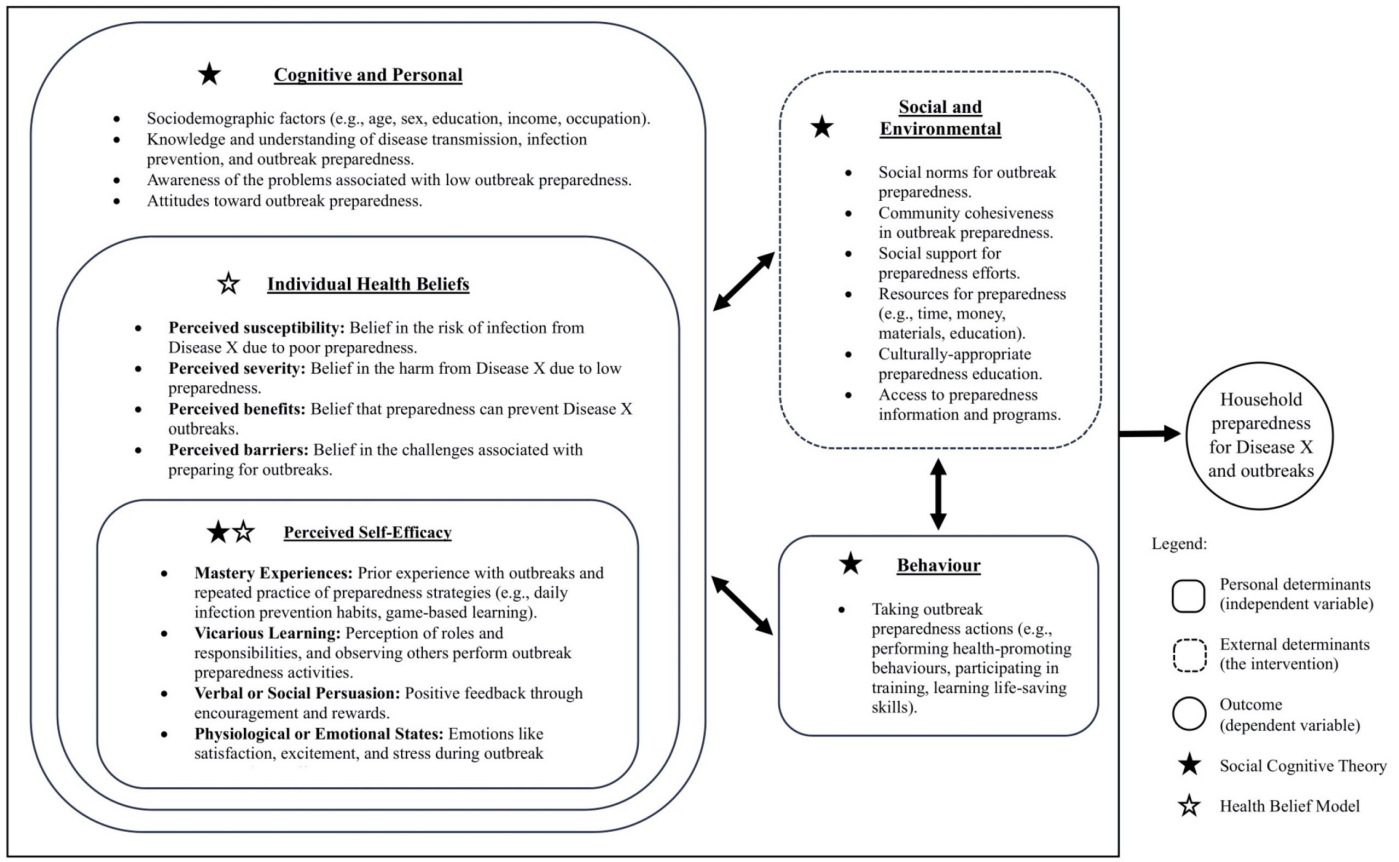
Theoretical framework.

**Figure 2:**
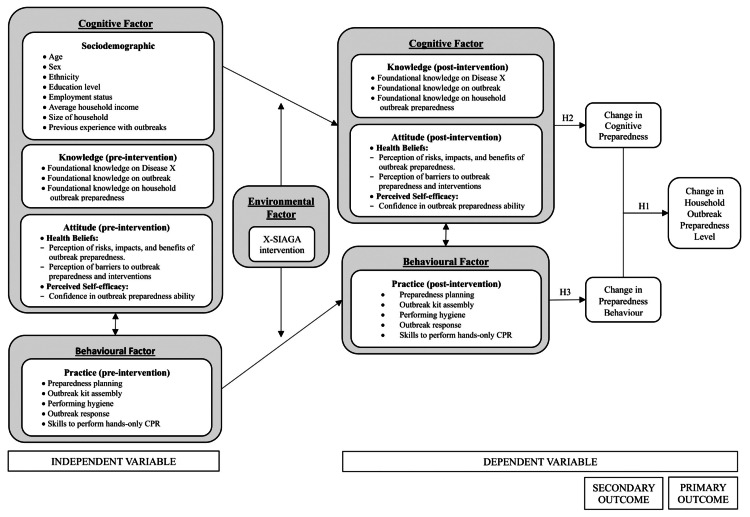
Conceptual framework. Hypothesis 1 (H1): The intervention changes household preparedness levels, potentially improving their capability to respond to Disease X and potential outbreaks; Hypothesis 2 (H2): The intervention changes cognitive aspects related to preparedness, improving knowledge and attitudes, including health beliefs, towards Disease X and outbreaks; Hypothesis 3 (H3): The intervention changes behavioural aspects related to preparedness, enhancing skills and tangible preparedness practices among households for Disease X and outbreak preparedness.

In this study, Cognitive Preparedness refers to the mental process of preparedness, specifically the knowledge, attitudes, and health beliefs a person holds regarding readiness for Disease X and outbreaks. Preparedness Behavior refers to the observable actions or practices carried out in preparation for such events. Literature suggests that effective preparedness requires both cognitive and behavioral components. Individuals need to have sufficient knowledge and positive attitudes and health beliefs, and they must also demonstrate these through actual preparedness behaviors [12,13]. Therefore, *Household Outbreak Preparedness* is defined as a continuum that integrates both *Cognitive Preparedness* and *Preparedness Behavior* to best reflect the overall level of preparedness [24]. The theoretical framework is presented in Figure 1, and the conceptual framework is presented in Figure 2.

Study setting and participants

The trial will take place in Orang Asli settlements in Selangor that are gazetted and listed under the Department of Orang Asli Development (JAKOA), focusing on the Orang Asli community members as the study population.

Recruitment

For each selected district, a JAKOA representative is engaged to support the study. The JAKOA representative contacts the village leader of eligible villages to gauge interest in participating. If the village leader expresses interest, an initial meeting is scheduled on a mutually agreed date involving JAKOA, the village leader and committee members, and the research team. During this meeting, the study is explained in detail, and any questions or concerns are addressed. Once the village leader and committee members express their commitment, they are provided with a study information sheet, eligibility criteria, participant expectations, and an ethical information statement.

The study is then promoted by the village leader and committee members to eligible participants. A list of interested and eligible individuals is shared with the research team. The research team prepares a WhatsApp invitation, which is sent to and forwarded by the village leader to the listed participants. Participants who attend the baseline assessment on the agreed date and time are briefed on the study and the consent process. Those who provide written informed consent will complete a baseline assessment and follow their cluster’s assigned intervention. Data collection will commence after participants have signed the informed consent forms.

Sample size

Using the formula for a two-arm parallel-group design in cluster trials [25], the trial requires a minimum sample size of 30 households for each arm. The calculations are as follows:

$m = \frac{(Z_{1-\alpha/2} + Z_{1-\beta})^2 \, 2\sigma^2}{\Delta^2} \left[ 1 + (n - 1)\rho \right]$

where *m* is the number of households per arm; *Z_1−α/2_* is the standard error associated with a 95% confidence interval (*Z_1−α/2_*=1.96); *Z_1−β_* is the standard error associated with 80% power (*Z_1−β_*=0.842); Δ is the mean awareness score difference between the pre- and post-assessments in the intervention group within a community-based trial on disaster preparedness intervention (Δ=15.97) [26]; σ^2^ is the total variation in awareness score between the pre- and post-assessments in the intervention group within the same trial (σ^2^=138.949) [26]; *n* is the estimated median number of households in Proto-Malay majority Orang Asli villages in Selangor (*n*=54) [18]; and ρ is the intracluster correlation coefficient (ICC) (ρ=0.048) [27].

Allowing for an anticipated attrition rate of approximately 30%, the ideal sample size was set at 45 households per arm. Following the CONSORT Statement recommendation that each study arm should include at least four clusters [28], this study employs multistage sampling to select four Orang Asli villages per arm, aiming for 12 households in each village, a total of 96 households for the study.

Eligibility criteria

Table 2 outlines the eligibility criteria for clusters at both the district and village levels. Meanwhile, Table 3 provides the inclusion and exclusion criteria for participants who will represent their households by participating in X-SIAGA and completing a questionnaire designed to assess the change in preparedness scores at all data collection points.

**Table 2:** Eligibility criteria for district and village level clusters.

District	Village
Districts in the state of Selangor.	Villages that have not previously participated in any outbreak preparedness programs.
Districts with gazetted Orang Asli villages.	Villages with more than 12 households.
Districts with more than 5 Orang Asli villages.	Villages accessible via road transport.

**Table 3:** Inclusion and exclusion criteria of participants.

Inclusion criteria	Exclusion criteria
Orang Asli descent.	Having physical or cognitive disabilities (e.g., blindness, deafness, schizophrenia and dementia).
Malaysian citizen.	Having severe health conditions (e.g., respiratory and cardiovascular disease requiring oxygen support such as severe asthma, Chronic Obstructive Pulmonary Disease, heart failure and congenital heart diseases).
Age 18 years old and above.	Residing temporarily (less than 2 years).
The head of the household, or the person who contributes to, or influences health-related decisions for the household.	Intending to relocate to a different village within the next 2 years.
Live in a household with more than two members.	
Able to converse, read and write in Malay.	

Sampling, randomization, allocation, and blinding

The study utilizes a multistage sampling approach with computer-generated randomization for the allocation of villages into the intervention or control arm. Due to potential variability in participant numbers and logistical challenges, simple randomization is employed over block randomization for its flexibility and straightforward, unbiased allocation. The entire sampling, randomization, and allocation process is conducted independently by an external health professional with research experience.

First, two districts in Selangor are randomly selected from the eligible pool. Within each district, four villages are randomly chosen. These villages are then divided, with two randomly allocated to the intervention group and two to the control group in each district (i.e., each district has two intervention villages and two control villages). This approach aims to achieve homogeneous sociodemographic groups among the households. Household recruitment in each village is voluntary, facilitated by JAKOA, village leaders, and committees, targeting 12 households per village.

Given the nature of the intervention, the authors are inevitably aware of group allocation. Procedures are identical for both groups, and research assistants are not provided with information about the study sequence or allocation; hence, they remain uninformed about group assignment. Participants in both groups will remain blinded to their allocation throughout the study.

Intervention

The X-SIAGA intervention package was developed by using the Intervention Mapping framework, and its development process will be reported in a separate publication. Table 4 provides a concise overview of the components and activities included in the X-SIAGA intervention. In the intervention group, households from four Orang Asli villages will receive educational brochures on communicable disease prevention and participate in the X-SIAGA intervention during a one-day program in their respective villages. Two eligible adults from each household will participate in the intervention activities.

**Table 4:** Summary of the components and contents of X-SIAGA intervention package.

Component	Contents
Lecture	Introduction to Disease X and outbreak.
	Importance of Household Outbreak Preparedness.
Video presentation	Video presentation on previous outbreaks and discussion.
Experience sharing	Sharing personal experiences with past outbreaks and discussion.
Practical session	Pre-Outbreak: (i) Infection prevention practice: Practicing the 7-step hand hygiene; (ii) Preparedness planning: Developing a household preparedness plan for outbreaks and Disease X.
	During and post-outbreak: (i) Suspecting an outbreak: Recognizing potential symptoms of Disease X and signs of outbreak through a puzzle game; (ii) Outbreak response: Learning what to do in the event of an outbreak through a sequencing game; (iii) Life-saving skills for emergency situations: Performing Hands-Only Cardiopulmonary Resuscitation (CPR).
Simulation exercise	Role-playing three different possible real-life situations: (i) Pre-Outbreak scenario; (ii) During outbreak scenario; (iii) Hands-only CPR scenario.
Game-based learning	“Wabak X” card game play session.

The intervention will be delivered by the authors, assisted by a team of research assistants who are independent of any institutions associated with the authors. All research assistants received prior training and were actively involved at an early stage, including during the pilot phase of the trial.

Each household will also receive a booklet that includes the materials covered during the X-SIAGA program. Participants start using the booklet during the program, and they take it home for self-study with other family members and to complete their household outbreak preparedness plans. Additionally, they will receive a set of the “Wabak X” card game and are encouraged to play it with their family members. The pair of adults representing their household will complete a questionnaire to assess the change in preparedness scores at all data collection points.

This study aims to assess the effectiveness of X-SIAGA under realistic conditions. This means there will be no refresher sessions or reminders. Instead, only follow-ups at specific time points for data collection will occur. This approach is designed to mirror real-world conditions, as health promotion programs conducted by various local agencies, including government ministries and community organizations, are typically held only once or at most twice per year.

Control

In the control group, households from four Orang Asli villages receive only educational brochures on communicable disease prevention, without the X-SIAGA intervention package. These brochures, sourced from the District Health Office, are currently used by the public health promotion team for education on awareness and prevention of infectious diseases and outbreaks in local communities. Similarly, two eligible adults from each control group household will participate in the research activities and complete the questionnaire at all data collection points. The control group will receive the X-SIAGA intervention package after data collection for all participants is completed.

Data collection

Data collection will be conducted by using the Household Outbreak Preparedness Evaluation (HOPE) questionnaire, administered at four intervals: (i) before the intervention (T0); (ii) immediately after the intervention, with the intervention group having received both the X-SIAGA intervention and educational brochures and the control group having received educational brochures only (T1); (iii) at one month post-intervention (T2); and (iv) at three months post-intervention (T3). The HOPE questionnaire is an adapted and translated instrument based on prior studies on household preparedness for outbreaks and general disasters [15,24]. Validation of the HOPE questionnaire will be published elsewhere. The questionnaire is self-administered, with assistance available from trained research assistants and representatives of the village committee if participants require support.

Outcome measures

The scoring for the HOPE questionnaire follows Sumaja’s methods [24]. The primary outcome measure, the Household Outbreak Preparedness score, represents the equally weighted average of the Cognitive Preparedness and Preparedness Behavior scores. By using Sumaja’s preparedness matrix [24], scores will be normalized to a scale of zero to one, and a cut-off value of 0.5 at the x- and y-axes will categorize households’ outbreak preparedness into four groups: Fully Prepared, Committed, Aware, and Unprepared.

Secondary outcome measures include the Cognitive Preparedness and Preparedness Behavior scores. The Cognitive Preparedness score assesses household knowledge, attitudes, and health beliefs, while the Preparedness Behavior score evaluates the skills acquired and practices adopted by households to prepare for potential Disease X and outbreaks.

Control of bias

To prevent contamination bias, the study ensures sufficient geographical separation between intervention and control villages to minimize interactions among participants from different groups. Prior to recruitment, the research team held meetings with village leaders and committee members, in the presence of JAKOA officials, to explain the X-SIAGA intervention, emphasize the importance of strict confidentiality, and discourage discussions about the intervention. These leaders and committee members then conveyed this information to eligible participants during household recruitment. All participants will again receive a detailed explanation of the privacy and confidentiality policy when expressing interest in joining the trial, and only those who provide consent will be enrolled. To mitigate attrition bias, the study will employ an Intention-to-Treat (ITT) approach. Additionally, to sustain motivation and engagement, perishable goods will be offered as incentives at each stage of data collection.

Statistical methods

The statistical analysis for this study will be carried out by using the IBM SPSS Statistics for Windows, Version 26 (Released 2018; IBM Corp., Armonk, New York). Descriptive statistics will include frequency and percentages for categorical data and mean and standard deviation for numerical data.

To compare baseline sociodemographic characteristics between the intervention and control groups, a Pearson Chi-Square test will be utilized for categorical variables, while an Independent Samples t-test will be used for continuous variables. To evaluate changes over time from T0 to T3 between and within the groups, a repeated-measures analysis of variance will be conducted. To assess variation in results between and within the groups across the different time points, while also controlling for potential confounding variables, a generalized estimating equation will be utilized.

Statistical analyses will use a two-tailed test to evaluate the superiority hypothesis. The null hypothesis states that there is no difference between the X-SIAGA intervention package and standard educational brochures. A significance level of 0.05 will be applied.

Data management

To manage missing data and outliers, corrective actions will be taken to recover or validate entries. The research team will reach out to the participant for clarification and ensure accurate data entry. The Last Observation Carried Forward method will be utilized for uncontactable dropouts. A sensitivity analysis will be performed to determine how using the ITT approach with imputed data might affect study conclusions.

During the research, data will be managed carefully to ensure privacy and confidentiality. All data generated will be de-identified and anonymous, with the principal investigator being the only one with access to identifiable information. Results will be presented in aggregated form to prevent reidentification of any individual or address. The findings from this study will be disseminated as conference abstracts, posters, and presentations, and manuscripts will be developed for publication from the study findings.

Individual participant data will be shared only upon reasonable request and will require approval from UiTM and the Ministry of Health Malaysia (MOH). These datasets will not contain any identifiers. Participants may request access to their own results and to the general study findings, but not to other participants’ data.

All data will be stored in soft-copy format on a password-protected computer and archived on a secure, password-protected flash drive to prevent data loss or damage. Data will be retained for seven years after publication, after which it will be securely destroyed by deleting and overwriting the files.

Trial registration, ethics approval, and consent to participate

This study has been registered at ClinicalTrials.gov (NCT06539845) and has received ethical approval from the National Medical Research Registry/Medical Research and Ethics Committee (Ref: 24-01859-NQ7) and the Universiti Teknologi MARA Research Ethics Committee (Ref: REC/10/2024 (PG/FB/33)).

This study adheres to the Malaysian Code of Responsible Conduct in Research to ensure data confidentiality and compliance with ethical standards. Engagement with the indigenous community throughout the study is conducted in a manner that is mutually beneficial, respectful, and sensitive to cultural norms. Participants are provided with a study information sheet that offers detailed information about the study’s purpose, procedures, and what participation entails. This information is given during recruitment advertisements and again before participants give consent.

Consent will be secured through written consent forms from participants before joining the study. Informed consent will be obtained by the research team, with assistance from representatives of village committee members provided as needed to ensure full understanding of the information. Investigators are available to answer any questions, and ample time is given for consideration of involvement in the study. All prospective participants are informed that participation is entirely voluntary. Withdrawal from the study is permitted at any time without any negative consequences, and there is no requirement to disclose reasons for withdrawal.

## Results

Results are not yet available at this stage. Data will be compiled in Microsoft Excel (Microsoft Corporation, Redmond, Washington) and analyzed using IBM SPSS as outlined in the Materials and Methods section. The analysis and reporting of results are planned upon study completion. The results of the study will be reported in accordance with the Consolidated Standards of Reporting Trials (CONSORT) statement [28]. It is anticipated that the intervention will lead to improved preparedness levels among participating households.

## Discussion

This study will be a two-arm cluster randomized trial evaluating the effectiveness of the X-SIAGA intervention. The X-SIAGA intervention is a novel, theory-based intervention package tailored for the Orang Asli community to enhance their household preparedness for Disease X and future outbreaks. This intervention aims to empower the community to respond effectively to infectious disease outbreaks, including novel threats, while waiting for intervention from health authorities, thereby reducing the negative impact and mortality rates associated with such events.

X-SIAGA addresses a crucial gap in micro-level preparedness. While numerous initiatives focus on macro-level preparedness, outbreaks invariably begin and spread within communities before being detected by authorities. Marginalized populations, such as Indigenous communities, are often disproportionately affected due to pre-existing vulnerabilities and limited access to resources [17,19]. Most existing community preparedness programs in Malaysia target floods, with no known initiatives addressing outbreak preparedness at the household level, especially among the Orang Asli [9]. X-SIAGA is pioneering as the first outbreak preparedness program in Malaysia tailored to the needs of the Orang Asli.

The study’s findings are anticipated to provide essential insights and evidence for the development of targeted health interventions, significantly contributing to enhancing public health preparedness and resilience, especially within marginalized communities.

While X-SIAGA has its strengths, there are notable limitations to the study. The expectation of immediate personal benefits among the Orang Asli population may pose challenges in participant recruitment and retention. The potential Hawthorne effect cannot be fully dismissed; mitigation efforts will include assuring participants that responses will not incur any penalties.

The X-SIAGA intervention will be conducted in a single session, with a follow-up period restricted to three months due to resource and time constraints. While some studies have shown that community interventions delivered in one session can lead to beneficial outcomes in a short timeframe [29,30], expecting sustained change from a single session may not be realistic, and short-term follow-ups might overlook the long-term effects of the intervention.

The generalizability of the findings might be limited due to the unique aspects of the Orang Asli community in Malaysia. The Orang Asli community in Selangor may also not be representative of Orang Asli tribes from other states, as some can be very different from one another. Furthermore, nonrandom household recruitment could lead to homogeneous views within social networks, and individuals holding village roles or being more vocal might disproportionately influence the study’s findings.

## Conclusions

This protocol outlines X-SIAGA, an intervention package designed to enhance preparedness for Disease X and potential outbreaks among Orang Asli households in Selangor. By comparing X-SIAGA with conventional educational brochures, the study aims to determine whether this comprehensive, tailored, and evidence-based intervention provides measurable benefits. If proven effective, X-SIAGA could be proposed for wider implementation and adapted for use in other communities.
